# Supplementary material for: SARS-CoV-2 Omicron is an immune escape variant with an altered cell entry pathway
Source: Nat Microbiol. 2022 Jul 7;7(8):1161–79. doi: 10.1038/s41564-022-01143-7 (PMC9352574; doi:10.1038/s41564-022-01143-7)
Supplement: Supplementary file 1 — Supplementary Tables 1–9. [file 41564_2022_1143_MOESM1_ESM.pdf]

nature portfolio

https://doi.org/10.1038/s41564-022-01143-7

Supplementary information

SARS-CoV-2 Omicron is an immune escape variant with an altered cell entry pathway

In the format provided by the authors and unedited

SARS-CoV-2 Omicron is an immune escape variant with an altered cell entry pathway

Brian J. Willett, Joe Grove, Oscar A. MacLean, Craig Wilkie, Giuditta De Lorenzo, Wilhelm Furnon, Diego Cantoni, Sam Scott, Nicola Logan, Shirin Ashraf, Maria Manali, Agnieszka Szemiel, Vanessa Cowton, Elen Vink, William T. Harvey, Chris Davis, Patawee Asamaphan, Katherine Smollett, Lily Tong, Richard Orton, Joseph Hughes, Poppy Holland, Vanessa Silva, David J. Pascall, Kathryn Puxty, Ana da Silva Filipe, Gonzalo Yebra, Sharif Shaaban, Matthew T. G. Holden, Rute Maria Pinto, Rory Gunson, Kate Templeton, Pablo R. Murcia, Arvind H. Patel, Paul Klenerman, Susanna Dunachie, PITCH Consortium*, The COVID-19 Genomics UK (COG-UK) Consortium*, John Haughney, David L. Robertson, Massimo Palmarini, Surajit Ray & Emma C. Thomson

Supplementary Tables 1 to 9

Supplementary Table 1. Comparison of SARS-CoV-2 antibody responses elicited by two doses of SARS-CoV-2 vaccine. Data were analyzed in GraphPad Prism v8.4.3, groups were compared by ordinary one-way ANOVA.

Tukey's multiple comparisons test	Mean Diff.	95.00% CI of diff.	Significant?	Summary	Adjusted P Value
Spike BNT162b2 vs. ChAdOx1	2588	-278.1 to 5453	No	ns	0.1212
Spike BNT162b2 vs. mRNA-1273	-248.4	-3114 to 2617	No	ns	>0.9999
Spike ChAdOx1 vs. mRNA-1273	-2836	-5702 to 29.73	No	ns	0.0553
RBD BNT162b2 vs. ChAdOx1	4649	1783 to 7514	**Yes**	****	**<0.0001**
RBD BNT162b2 vs. mRNA-1273	-449.1	-3315 to 2417	No	ns	>0.9999
RBD ChAdOx1 vs. mRNA-1273	-5098	-7964 to -2232	**Yes**	****	**<0.0001**
NTD BNT162b2 vs. ChAdOx1	5755	2889 to 8620	**Yes**	****	**<0.0001**
NTD BNT162b2 vs. mRNA-1273	714.7	-2151 to 3581	No	ns	0.9996
NTD ChAdOx1 vs. mRNA-1273	-5040	-7906 to -2174	**Yes**	****	**<0.0001**
N BNT162b2vs. ChAdOx1	2.183	-2864 to 2868	No	ns	>0.9999
N BNT162b2 vs. mRNA-1273	0.4	-2865 to 2866	No	ns	>0.9999
N ChAdOx1 vs. mRNA-1273	-1.783	-2868 to 2864	No	ns	>0.9999

Supplementary Table 2. Comparison of HCoV antibody responses elicited by two doses of SARS-CoV-2 vaccine. Data were analyzed in GraphPad Prism v8.4.3, groups were compared by ordinary one-way ANOVA.

Tukey's multiple comparisons test	Mean Diff.	95.00% CI of diff.	Significant?	Summary	Adjusted P Value
229E BNT162b2 vs. ChAdOx1	3296	-19673 to 26265	No	ns	>0.9999
229E BNT162b2 vs. mRNA-1273	5282	-17687 to 28251	No	ns	0.9998
229E ChAdOx1 vs. mRNA-1273	1986	-20982 to 24955	No	ns	>0.9999
OC43 BNT162b2 vs. ChAdOx1	39581	16612 to 62550	Yes	****	<0.0001
OC43 BNT162b2 vs. mRNA-1273	18396	-4573 to 41365	No	ns	0.2628
OC43 ChAdOx1 vs. mRNA-1273	-21185	-44154 to 1784	No	ns	0.1027
NL63 BNT162b2 vs. ChAdOx1	1317	-21652 to 24286	No	ns	>0.9999
NL63 BNT162b2 vs. mRNA-1273	696.9	-22272 to 23666	No	ns	>0.9999
NL63 ChAdOx1 vs. mRNA-1273	-620	-23589 to 22349	No	ns	>0.9999
HKU1 BNT162b2vs. ChAdOx1	17914	-5055 to 40883	No	ns	0.3015
HKU1 BNT162b2 vs. mRNA-1273	5468	-17501 to 28437	No	ns	0.9998
HKU1 ChAdOx1 vs. mRNA-1273	-12446	-35415 to 10523	No	ns	0.8243

Supplementary Table 3. Comparison of influenza antibody responses elicited by two doses of SARS-CoV-2 vaccine. Data were analyzed in GraphPad Prism v8.4.3, groups were compared by ordinary one-way ANOVA.

Tukey's multiple comparisons test	Mean Diff.	95.00% CI of diff.	Significant?	Summary	Adjusted P Value
Flu A Michigan H1 BNT162b2 vs. ChAdOx1	42355	-49090 to 133800	No	ns	0.9615
Flu A Michigan H1 BNT162b2 vs. mRNA-1273	34380	-57065 to 125825	No	ns	0.9943
Flu A Michigan H1 ChAdOx1 vs. mRNA-1273	-7975	-99420 to 83469	No	ns	>0.9999
Flu A Hong Kong H3 BNT162b2 vs. ChAdOx1	22853	-68592 to 114298	No	ns	>0.9999
Flu A Hong Kong H3 BNT162b2 vs. mRNA-1273	43959	-47486 to 135403	No	ns	0.948
Flu A Hong Kong H3 ChAdOx1 vs. mRNA-1273	21106	-70339 to 112550	No	ns	>0.9999
Flu A Shanghai H7 BNT162b2 vs. ChAdOx1	-3370	-94815 to 88074	No	ns	>0.9999
Flu A Shanghai H7 BNT162b2 vs. mRNA-1273	-5570	-97015 to 85874	No	ns	>0.9999
Flu A Shanghai H7 ChAdOx1 vs. mRNA-1273	-2200	-93645 to 89244	No	ns	>0.9999
Flu B Phuket HA BNT162b2 vs. ChAdOx1	54701	-36744 to 146145	No	ns	0.7707
Flu B Phuket HA BNT162b2 vs. mRNA-1273	83834	-7611 to 175279	No	ns	0.1132
Flu B Phuket HA ChAdOx1 vs. mRNA-1273	29133	-62311 to 120578	No	ns	0.999
Flu B Brisbane BNT162b2 vs. ChAdOx1	35122	-56323 to 126566	No	ns	0.993
Flu B Brisbane BNT162b2 vs. mRNA-1273	64342	-27102 to 155787	No	ns	0.5138
Flu B Brisbane ChAdOx1 vs. mRNA-1273	29220	-62224 to 120665	No	ns	0.999

Supplementary Table 4. Comparison of neutralising antibody titres elicited by two doses of SARS-CoV-2 vaccine. Neutralising antibody responses were quantified against Wuhan or Omicron spike glycoprotein-bearing HIV(SARS-CoV-2) pseudotypes. Data were analyzed in GraphPad Prism v8.4.3, groups were compared by ordinary one-way ANOVA.

Tukey's multiple comparisons test	Mean 1	Mean 2	Mean Diff.	95.00% CI of diff.	Significant?	Summary	Adjusted P Value
B.1 BNT162b2 vs. ChAdOx1	4978	882.3	4096	733.8 to 7458	Yes	**	0.0075
B.1 BNT162b2 vs. mRNA-1273	4978	21118	-16140	-19502 to -12778	Yes	****	<0.0001
B.1 ChAdOx1 vs. mRNA-1273	882.3	21118	-20236	-23598 to -16874	Yes	****	<0.0001
BA.1 BNT162b2 vs. ChAdOx1	148.3	61.9	86.39	-3276 to 3448	No	ns	>0.9999
BA.1 BNT162b2 vs. mRNA-1273	148.3	285	-136.7	-3499 to 3225	No	ns	>0.9999
BA.1 ChAdOx1 vs. mRNA-1273	61.9	285	-223.1	-3585 to 3139	No	ns	>0.9999
BNT162b2 B.1 vs. BA.1	4978	148.3	4830	1468 to 8192	Yes	***	0.0008
ChAdOx12 B.1 vs. BA.1	882.3	61.9	820.4	-2542 to 4182	No	ns	0.981
mRNA-1273 B.1 vs. BA.1	21118	285	20833	17471 to 24195	Yes	****	<0.0001

Supplementary Table 5. Comparison of SARS-CoV-2 antibody responses elicited by a third dose of SARS-CoV-2 vaccine. Data were analyzed in GraphPad Prism v8.4.3, groups were compared by ordinary one-way ANOVA. P= BNT162b2, AZ = ChAdOx1, M = mRNA-1273.

Tukey's multiple comparisons test	Mean Diff.	95.00% CI of diff.	Significant?	Summary	Adjusted P Value
Spike AZ+P vs. AZ+M	1163	-4354 to 6680	No	ns	>0.9999
Spike AZ+P vs. P+P	1007	-3360 to 5374	No	ns	>0.9999
Spike AZ+P vs. P+M	361.3	-8410 to 9133	No	ns	>0.9999
Spike AZ+M vs. P+P	-156	-5239 to 4927	No	ns	>0.9999
Spike AZ+M vs. P+M	-801.5	-9951 to 8348	No	ns	>0.9999
Spike P+P vs. P+M	-645.5	-9151 to 7860	No	ns	>0.9999
RBD AZ+P vs. AZ+M	2675	-2842 to 8192	No	ns	0.9453
RBD AZ+P vs. P+P	1439	-2928 to 5806	No	ns	0.9988
RBD AZ+P vs. P+M	37.5	-8734 to 8809	No	ns	>0.9999
RBD AZ+M vs. P+P	-1236	-6319 to 3846	No	ns	>0.9999
RBD AZ+M vs. P+M	-2638	-11787 to 6511	No	ns	0.9997
RBD P+P vs. P+M	-1401	-9906 to 7104	No	ns	>0.9999
NTD AZ+P vs. AZ+M	3368	-2149 to 8885	No	ns	0.7418
NTD AZ+P vs. P+P	4098	-269.4 to 8465	No	ns	0.0919
NTD AZ+P vs. P+M	3739	-5033 to 12510	No	ns	0.9822
NTD AZ+M vs. P+P	729.8	-4353 to 5813	No	ns	>0.9999
NTD AZ+M vs. P+M	370.8	-8778 to 9520	No	ns	>0.9999
NTD P+P vs. P+M	-359	-8864 to 8146	No	ns	>0.9999
N AZ+P vs. AZ+M	2.755	-5514 to 5520	No	ns	>0.9999
N AZ+P vs. P+P	-0.7066	-4368 to 4366	No	ns	>0.9999
N AZ+P vs. P+M	-3.095	-8775 to 8768	No	ns	>0.9999
N AZ+M vs. P+P	-3.461	-5086 to 5079	No	ns	>0.9999
N AZ+M vs. P+M	-5.85	-9155 to 9143	No	ns	>0.9999
N P+P vs. P+M	-2.389	-8508 to 8503	No	ns	>0.9999

P = BNT162b2; AZ = ChAdOx1; M = mRNA-1273

Supplementary Table 6. Comparison of HCoV antibody responses elicited by a third dose of SARS-CoV-2 vaccine. Data were analyzed in GraphPad Prism v8.4.3, groups were compared by ordinary one-way ANOVA. P= BNT162b2, AZ = ChAdOx1, M = mRNA-1273.

Tukey's multiple comparisons test	Mean Diff.	95.00% CI of diff.	Significant?	Summary	Adjusted P Value
229E AZ+P vs. AZ+M	-11229	-47020 to 24562	No	ns	0.9993
229E AZ+P vs. P+P	-5670	-34000 to 22661	No	ns	>0.9999
229E AZ+P vs. P+M	4487	-52417 to 61391	No	ns	>0.9999
229E AZ+M vs. P+P	5560	-27414 to 38533	No	ns	>0.9999
229E AZ+M vs. P+M	15716	-43637 to 75069	No	ns	>0.9999
229E P+P vs. P+M	10156	-45019 to 65332	No	ns	>0.9999
OC43 AZ+P vs. AZ+M	-29568	-65359 to 6224	No	ns	0.2369
OC43 AZ+P vs. P+P	-121	-28451 to 28209	No	ns	>0.9999
OC43 AZ+P vs. P+M	-5301	-62205 to 51603	No	ns	>0.9999
OC43 AZ+M vs. P+P	29447	-3527 to 62420	No	ns	0.1382
OC43 AZ+M vs. P+M	24266	-35086 to 83619	No	ns	0.988
OC43 P+P vs. P+M	-5180	-60356 to 49996	No	ns	>0.9999
NL63 AZ+P vs. AZ+M	-11140	-46931 to 24651	No	ns	0.9994
NL63 AZ+P vs. P+P	-8074	-36404 to 20256	No	ns	0.9998
NL63 AZ+P vs. P+M	-16842	-73746 to 40062	No	ns	0.9996
NL63 AZ+M vs. P+P	3066	-29908 to 36040	No	ns	>0.9999
NL63 AZ+M vs. P+M	-5702	-65055 to 53651	No	ns	>0.9999
NL63 P+P vs. P+M	-8768	-63944 to 46407	No	ns	>0.9999
HKU1 AZ+P vs. AZ+M	9482	-26309 to 45273	No	ns	>0.9999
HKU1 AZ+P vs. P+P	8028	-20303 to 36358	No	ns	0.9998
HKU1 AZ+P vs. P+M	8678	-48226 to 65582	No	ns	>0.9999
HKU1 AZ+M vs. P+P	-1454	-34428 to 31519	No	ns	>0.9999
HKU1 AZ+M vs. P+M	-804.1	-60157 to 58549	No	ns	>0.9999
HKU1 P+P vs. P+M	650.4	-54525 to 55826	No	ns	>0.9999

P = BNT162b2; AZ = ChAdOx1; M = mRNA-1273

Supplementary Table 7. Comparison of influenza antibody responses elicited by a third dose of SARS-CoV-2 vaccine. Data were analyzed in GraphPad Prism v8.4.3, groups were compared by ordinary one-way ANOVA. P= BNT162b2, AZ = ChAdOx1, M = mRNA-1273.

Tukey's multiple comparisons test	Mean Diff.	95.00% CI of diff.	Significant?	Summary	Adjusted P Value
Flu A Michigan H1 AZ+P vs. AZ+M	410671	6882 to 814460	Yes	*	0.0413
Flu A Michigan H1 AZ+P vs. P+P	279008	-40609 to 598625	No	ns	0.1757
Flu A Michigan H1 AZ+P vs. P+M	459436	-182547 to 1101420	No	ns	0.5271
Flu A Michigan H1 AZ+M vs. P+P	-131663	-503667 to 240341	No	ns	0.9993
Flu A Michigan H1 AZ+M vs. P+M	48766	-620842 to 718373	No	ns	>0.9999
Flu A Michigan H1 P+P vs. P+M	180429	-442054 to 802911	No	ns	>0.9999
Flu A Hong Kong H3 AZ+P vs. AZ+M	-64025	-467814 to 339763	No	ns	>0.9999
Flu A Hong Kong H3 AZ+P vs. P+P	-32804	-352421 to 286812	No	ns	>0.9999
Flu A Hong Kong H3 AZ+P vs. P+M	56571	-585413 to 698554	No	ns	>0.9999
Flu A Hong Kong H3 AZ+M vs. P+P	31221	-340783 to 403225	No	ns	>0.9999
Flu A Hong Kong H3 AZ+M vs. P+M	120596	-549012 to 790204	No	ns	>0.9999
Flu A Hong Kong H3 P+P vs. P+M	89375	-533107 to 711857	No	ns	>0.9999
Flu A Shanghai H7 AZ+P vs. AZ+M	6841	-396948 to 410630	No	ns	>0.9999
Flu A Shanghai H7 AZ+P vs. P+P	469.8	-319147 to 320087	No	ns	>0.9999
Flu A Shanghai H7 AZ+P vs. P+M	9363	-632620 to 651346	No	ns	>0.9999
Flu A Shanghai H7 AZ+M vs. P+P	-6371	-378375 to 365633	No	ns	>0.9999
Flu A Shanghai H7 AZ+M vs. P+M	2522	-667086 to 672130	No	ns	>0.9999
Flu A Shanghai H7 P+P vs. P+M	8893	-613589 to 631376	No	ns	>0.9999
Flu B Phuket HA AZ+P vs. AZ+M	-101482	-505271 to 302307	No	ns	>0.9999
Flu B Phuket HA AZ+P vs. P+P	-6309	-325926 to 313308	No	ns	>0.9999
Flu B Phuket HA AZ+P vs. P+M	65660	-576323 to 707643	No	ns	>0.9999
Flu B Phuket HA AZ+M vs. P+P	95173	-276831 to 467178	No	ns	>0.9999
Flu B Phuket HA AZ+M vs. P+M	167142	-502466 to 836750	No	ns	>0.9999
Flu B Phuket HA P+P vs. P+M	71969	-550513 to 694451	No	ns	>0.9999
Flu B Brisbane AZ+P vs. AZ+M	-2954	-406743 to 400834	No	ns	>0.9999
Flu B Brisbane AZ+P vs. P+P	24661	-294956 to 344278	No	ns	>0.9999
Flu B Brisbane AZ+P vs. P+M	122461	-519522 to 764444	No	ns	>0.9999
Flu B Brisbane AZ+M vs. P+P	27615	-344389 to 399620	No	ns	>0.9999
Flu B Brisbane AZ+M vs. P+M	125415	-544193 to 795023	No	ns	>0.9999
Flu B Brisbane P+P vs. P+M	97800	-524683 to 720282	No	ns	>0.9999

P = BNT162b2; AZ = ChAdOx1; M = mRNA-1273

Supplementary Table 8. Effect of third dose of SARS-CoV-2 vaccine on neutralising antibody titres. Neutralising antibody responses were quantified against Wuhan or Omicron spike glycoprotein-bearing HIV (SARS-CoV-2) pseudotypes. Data were analyzed in GraphPad Prism v8.4.3, groups were compared by ordinary one-way ANOVA.

BNT162b2 prime (doses 1 & 2)					
**Tukey's multiple comparisons test**	**Mean Diff.**	**95.00% CI of diff.**	**Significant?**	**Summary**	**Adjusted P Value**
B.1 (dose 2) vs. (dose 3)	-1186	-4381 to 2010	No	ns	0.7655
BA.1 (dose 2) vs. (dose 3)	56.56	-3139 to 3252	No	ns	>0.9999
**Test details**	**Mean 1**	**Mean 2**	**Mean Diff.**	**SE of diff.**	**n1**
B.1 (dose 2) vs. (dose 3)	4978	6164	-1186	1219	24
BA.1 (dose 2) vs. (dose 3)	148.3	91.73	56.56	1219	24

ChAdOx1 prime (doses 1 & 2)					
**Tukey's multiple comparisons test**	**Mean Diff.**	**95.00% CI of diff.**	**Significant?**	**Summary**	**Adjusted P Value**
B.1 (dose 2) vs. (dose 3)	-7360	-11073 to -3647	**Yes**	****	**<0.0001**
BA.1 (dose 2) vs. (dose 3)	-203.7	-3916 to 3509	No	ns	0.9989
**Test details**	**Mean 1**	**Mean 2**	**Mean Diff.**	**SE of diff.**	**n1**
B.1 (dose 2) vs. (dose 3)	882.3	8242	-7360	1416	24
BA.1 (dose 2) vs. (dose 3)	61.9	265.5	-203.7	1416	24

Dose 3 titre comparison					
**Tukey's multiple comparisons test**	**Mean Diff.**	**95.00% CI of diff.**	**Significant?**	**Summary**	**Adjusted P Value**
B.1 (BNT162b2 dose 2) vs. (ChAdOX1 dose 2)	-2079	-7008 to 2851	No	ns	0.6859
BA.1 (BNT162b2 dose 2) vs. (ChAdOX1 dose 2)	-173.8	-5103 to 4756	No	ns	0.9997
**Test details**	**Mean 1**	**Mean 2**	**Mean Diff.**	**SE of diff.**	**n1**
B.1 (BNT162b2 dose 2) vs. (ChAdOX1 dose 2)	6164	8242	-2079	1877	20
BA.1 (BNT162b2 dose 2) vs. (ChAdOX1 dose 2)	91.73	265.5	-173.8	1877	20

Supplementary Table 9: Demographics, SARS-CoV-2 positivity status and vaccination status for the study population. Population consisted of 116,089 people aged 18 and over, registered as living in NHS Greater Glasgow and Clyde and tested by PCR test for SARS-CoV-2 infection between 6th and 26th December 2021, split by SARS-CoV-2 variant status.

	Negative	*Delta*	*Omicron*
**Demographics**			
**Age on 31st October 2021**			
Minimum	18.77	18.85	18.77
1st Quartile	31.66	32.89	26.59
Median	44.32	41.99	35.41
Mean	46.53	42.89	38.77
3rd Quartile	58.55	52.05	49.39
Maximum	108.56	100.16	102.82
**Sex**			
Female	55611 (56.74%)	3022 (3.08%)	9298 (8.43%)
Male	36570 (37.31%)	2806 (2.86%)	8782 (7.96%)
**SIMD (2016) vigintile**			
Minimum	1.00	1.00	1.00
1st Quartile	3.00	3.00	4.00
Median	9.00	9.00	9.00
Mean	9.39	9.38	9.81
3rd Quartile	16.00	16.00	16.00
Maximum	20.00	20.00	20.00
Missing values	1748	119	341
**SARS-CoV-2 positivity status**			
**Previous confirmed SARS-CoV-2 infection status**			
No previous infection	79050 (80.66%)	5745 (5.86%)	16396 (14.87%)
Had previous infection	13131 (13.40%)	83 (0.08%)	1684 (1.53%)
**Vaccination status**			
**Vaccination schedule**			
Unvaccinated	695 (0.80%)	58 (1.18%)	140 (0.84%)
1 dose only	2514 (2.89%)	243 (4.93%)	574 (3.46%)
2 doses ChAdOx1	21171 (24.33%)	2066 (41.92%)	4489 (27.08%)
2 doses BNT162b2	27050 (31.09%)	1771 (35.94%)	6926 (41.79%)
2 doses mRNA-1273	7035 (8.09%)	371 (7.53%)	2174 (13.12%)
3rd dose BNT162b2	21381 (24.57%)	319 (6.47%)	1624 (9.80%)
3rd dose mRNA-1273	7158 (8.23%)	100 (2.03%)	648 (3.91%)
**Time (days) since most recent dose by variant and dose number**			

	Negative			*Delta*			*Omicron*		
	1	*2*	*3*	*1*	*2*	*3*	*1*	*2*	*3*
Minimum	14.00	14.00	14.00	15.00	17.00	14.00	14.00	14.00	14.00
1st Quartile	87.00	119.00	30.00	111.00	129.00	29.75	90.00	113.00	26.00
Median	152.00	154.00	45.00	158.00	157.00	45.00	151.00	139.00	42.00
Mean	150.13	156.67	44.41	159.80	158.98	46.03	142.68	146.19	42.44
3rd Quartile	192.00	189.00	60.00	200.50	184.00	62.00	180.75	172.00	59.00
Maximum	361.00	462.00	285.00	329.00	324.00	236.00	331.00	460.00	128.00
