# Supplementary material for: SARS-CoV-2 Omicron is an immune escape variant with an altered cell entry pathway
Source: Nat Microbiol. 2022 Jul 7;7(8):1161–79. doi: 10.1038/s41564-022-01143-7 (PMC9352574; doi:10.1038/s41564-022-01143-7)
Supplement: Supplementary file 2 — Reporting Summary [file 41564_2022_1143_MOESM2_ESM.pdf]

nature portfolio

Corresponding author(s): Emma Thomson, Brian Willett, Joe Grove

Last updated by author(s): 2022/04/28

Reporting Summary

Nature Portfolio wishes to improve the reproducibility of the work that we publish. This form provides structure for consistency and transparency in reporting. For further information on Nature Portfolio policies, see our Editorial Policies and the Editorial Policy Checklist.

Statistics

For all statistical analyses, confirm that the following items are present in the figure legend, table legend, main text, or Methods section.

n/a	Confirmed	
☐	☒	The exact sample size (*n*) for each experimental group/condition, given as a discrete number and unit of measurement
☐	☒	A statement on whether measurements were taken from distinct samples or whether the same sample was measured repeatedly
☐	☒	The statistical test(s) used AND whether they are one- or two-sided<br>*Only common tests should be described solely by name; describe more complex techniques in the Methods section.*
☐	☒	A description of all covariates tested
☐	☒	A description of any assumptions or corrections, such as tests of normality and adjustment for multiple comparisons
☐	☒	A full description of the statistical parameters including central tendency (e.g. means) or other basic estimates (e.g. regression coefficient) AND variation (e.g. standard deviation) or associated estimates of uncertainty (e.g. confidence intervals)
☐	☒	For null hypothesis testing, the test statistic (e.g. *F*, *t*, *r*) with confidence intervals, effect sizes, degrees of freedom and *P* value noted<br>*Give P values as exact values whenever suitable.*
☒	☐	For Bayesian analysis, information on the choice of priors and Markov chain Monte Carlo settings
☒	☐	For hierarchical and complex designs, identification of the appropriate level for tests and full reporting of outcomes
☒	☐	Estimates of effect sizes (e.g. Cohen's *d*, Pearson's *r*), indicating how they were calculated

Our web collection on statistics for biologists contains articles on many of the points above.

Software and code

Policy information about availability of computer code

Data collection	Anonymised data were made available for analysis on the NHS GGC SafeHaven platform (EVADE)
Data analysis	Statistical analysis for the vaccine effectiveness calculations was carried out in R version 4.0.5 on the NHS GGC SafeHaven platform. The R scripts are available in the GitHub repository (https://github.com/centre-for-virus-research/Omicron).

For manuscripts utilizing custom algorithms or software that are central to the research but not yet described in published literature, software must be made available to editors and reviewers. We strongly encourage code deposition in a community repository (e.g. GitHub). See the Nature Portfolio guidelines for submitting code & software for further information.

Data

Policy information about availability of data

All manuscripts must include a data availability statement. This statement should provide the following information, where applicable:

- Accession codes, unique identifiers, or web links for publicly available datasets
- A description of any restrictions on data availability
- For clinical datasets or third party data, please ensure that the statement adheres to our policy

The experimental data that support the findings of this study are included with the submission (neutralisation, ELISpot, entry data) but restrictions apply to the availability of clinical data, which were used under ethical approvals for the current study behind an NHS firewall, and so are not publicly available. Biological materials including cell lines are available on reasonable request from the authors. Clinical samples are restricted for use under the ethical approvals obtained for their use.

Field-specific reporting

Please select the one below that is the best fit for your research. If you are not sure, read the appropriate sections before making your selection.

☒ Life sciences ☐ Behavioural & social sciences ☐ Ecological, evolutionary & environmental sciences

For a reference copy of the document with all sections, see nature.com/documents/nr-reporting-summary-flat.pdf

Life sciences study design

All studies must disclose on these points even when the disclosure is negative.

Sample size	For evaluation of vaccine effectiveness, all data points for people over 18 years old and living in the NHS GGC area with a PCR test for COVID-19 carried out between 2021-12-06 and 2021-12-26 were included in the analysis (and with either sequencing information, ASP status or SGTF status) recorded, for positives). For neutralisation (DOVE), age-matched participants (24/group) were selected.
Data exclusions	Those with a vaccine listed other than ChAdOxl, BNT162b2 or mRNA-1273, and those with multiple vaccinations listed on the same day with different products, were removed from the study. Only those with a PCR test for COVID-19 carried out between 2021-12-06 and 2021-12-26 (and with either sequencing information, ASP status or SGTF status recorded, for positives) were included in the study, to avoid biases due to incorrect population baseline estimates. Those who were due to receive a new vaccine dose but did not were excluded, to avoid bias due to infection delaying vaccination. Those who received a new vaccine dose during the study period were also excluded.
Replication	In vitro studies were performed using multiple replicates, the number of which is specified in each independent figure, source data are provided separately.
Randomization	Allocation to case or control group was defined by COVID-19 PCR status. This non-random allocation was controlled for using data on age, sex and deprivation index (SIMD) quartile for each participant (for calculation of vaccine effectiveness.)
Blinding	Blinding was not carried out, due to the observational nature of the study, with no randomization required or possible.

Reporting for specific materials, systems and methods

We require information from authors about some types of materials, experimental systems and methods used in many studies. Here, indicate whether each material, system or method listed is relevant to your study. If you are not sure if a list item applies to your research, read the appropriate section before selecting a response.

Materials & experimental systems

n/a	Involved in the study	
☐	☒	Antibodies
☐	☒	Eukaryotic cell lines
☒	☐	Palaeontology and archaeology
☒	☐	Animals and other organisms
☐	☒	Human research participants
☒	☐	Clinical data
☒	☐	Dual use research of concern

Methods

n/a	Involved in the study	
☒	☐	ChIP-seq
☒	☐	Flow cytometry
☒	☐	MRI-based neuroimaging

Antibodies

Antibodies used	The plates were incubated for 18 hours at 37oC, 5% CO2. The plates were then washed with PBS and incubated with 1µg/ml biotin-labelled detection antibody (7-B6-1, Mabtech)
Validation	Documentation and validation of this commercial antibody can be found at: https://www.mabtech.com/products/anti-human-ifn-gamma-antibody-7-b6-1-biotinylated-3420-6

Eukaryotic cell lines

Policy information about cell lines

Cell line source(s)	Cells. Calu-3 cells ATCC #HTB-55 are human lung adenocarcinoma epithelial cells. Caco-2 (CVR cytology cell bank) are an immortalized cell line derived from human colorectal adenocarcinoma, primarily used as a model of the intestinal epithelial barrier. A549 cells (ATCC #CCL-185) a human alveolar adenocarcinoma line, were a generous gift from Prof. Ben Hale, validated by STR analysis (Eurofins)). A549 were modified to stably express human ACE-2 and TMPRSS2. Human embryonic kidney (HEK293T) cells were used in pseudotype production. African green monkey kidney cells (Vero) were used to

	propagate the reverse genetics-derived viruses. Baby Hamster Kidney clone 21 cells (BHK-21 ATCC #CCL-10, purchased from ATCC, Bethesda, MD) and Vero ACE-2 TMPRSS272 cells were used in the isolation of live Omicron SARS-CoV-2. All cell lines were maintained at 37°C and 5% CO2 in DMEM supplemented with 10% foetal bovine serum (FBS), except for Calu-3 cells which were supplemented with 20% FBS. Human reconstituted upper airway epithelium (Mucilair™, abbreviated hNECs in this manuscript) were purchased from Epithelix and maintained in Mucilair complete culture basal medium (Epithelix) at an air-liquid interface.<br><br>Generation of BHK-21 cell line expressing human ACE2 receptor. Lentiviral vectors encoding human ACE2 (GenBank NM_001371415.1) were produced as described previously72. BHK-21 cells were transduced with the ACE2-encoding lentivirus and selected in medium containing 200 µg/ml of hygromycin B. A pool of hygromycin-resistant cells, BHK-ACE2, was used in this study.<br><br>Generation of cell lines used for fusion assays. Retrovirus vectors were produced by transfecting HEK-293T cells with plasmid pQCXIP-GFP1-10 (Addgene #68715) or pQCXIP-BSR-GFP11 (Addgene #68716)42 and packaging vectors expressing MLV gal-pol and VSV-G using Lipofectamine 3000 (Invitrogen) according to manufacturer's instructions. Cell supernatants were harvested 24-48h post-transfection, pooled, clarified by centrifugation and filtered. One mL of each supernatant was used to transduce A549-Ace2-TMPRSS2 (AAT) cells72 in the presence of Polybrene (Merck). Two days post-transduction, the supernatant was replaced with selection medium (DMEM 10% FBS 1µg/mL puromycin) and cells incubated until complete death of the non-transduced control cells were observed. The resulting puromycin-resistant cells (termed AAT-GFP1-10 and AAT-BSR-GFP11) were used in fusion assays.
Authentication	Not authenticated
Mycoplasma contamination	All cell lines were screened for Mycoplasma contamination
Commonly misidentified lines<br>(See ICLAC register)	NA

Human research participants

Policy information about studies involving human research participants

Population characteristics	Participants in the DOVE study were recipients of either 1, 2 or 3 doses of ChAdOx1, BNT162b2 or mRNA-1273 vaccines. Volunteers with immunosuppression and those who had previously been diagnosed with COVID-19 infection were excluded from the study.
Recruitment	Participants in the DOVE study were age but not sex-matched and gave written informed consent to participate in the study.
Ethics oversight	All participants in the DOVE study gave written informed consent to take part in the study which was approved by the North-West Liverpool Central Research Ethics Committee (REC reference 21/NW/0073). Residual nasopharyngeal swabs of patients infected with Omicron were collected with biorepository ethical approval (NHS Lothian reference 20/ES/0061). Derogated ethical approval for the use of demographic data for the EVADE study was granted by the NHS GG&C SafeHaven committee (GSH/21/IM/001).

Note that full information on the approval of the study protocol must also be provided in the manuscript.
