# Supplementary figures and images for: SARS-CoV-2 Omicron is an immune escape variant with an altered cell entry pathway
Source: Nat Microbiol. 2022 Jul 7;7(8):1161–79. doi: 10.1038/s41564-022-01143-7 (PMC9352574; doi:10.1038/s41564-022-01143-7)

Raw blots images used in Fig.  
51

4-12% Bis-Tris gels ran in MOPS running buffer

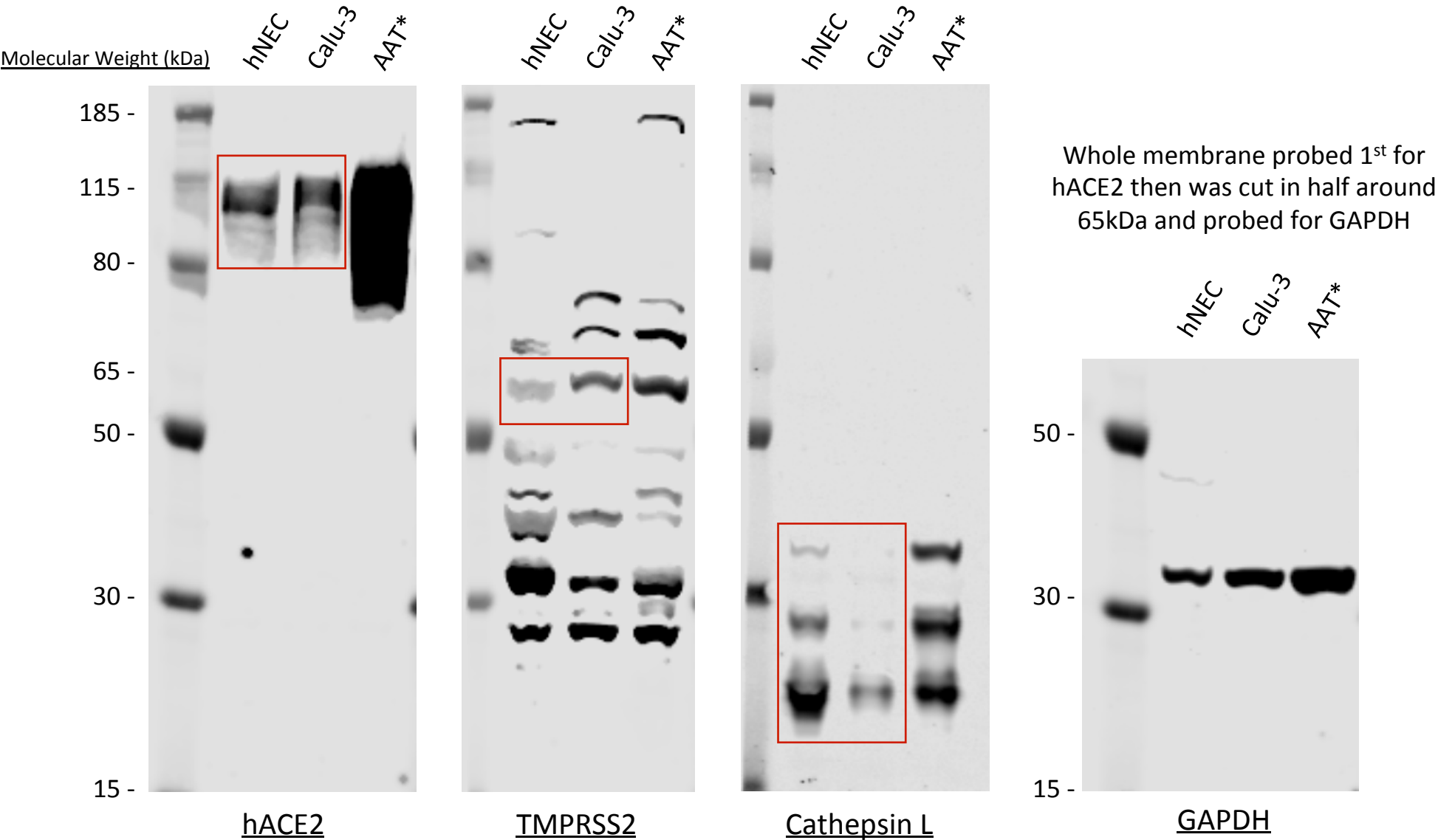

Supplement: Source Data Fig. 5 — Raw scans of blot for Fig. 5l. [file 41564_2022_1143_MOESM16_ESM.pdf]

Fig. 6 d

S1

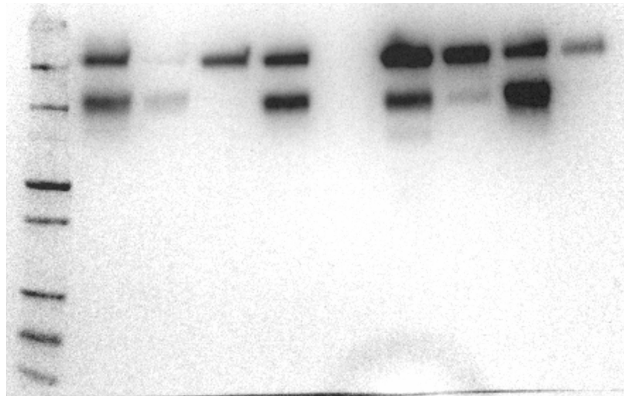

S2

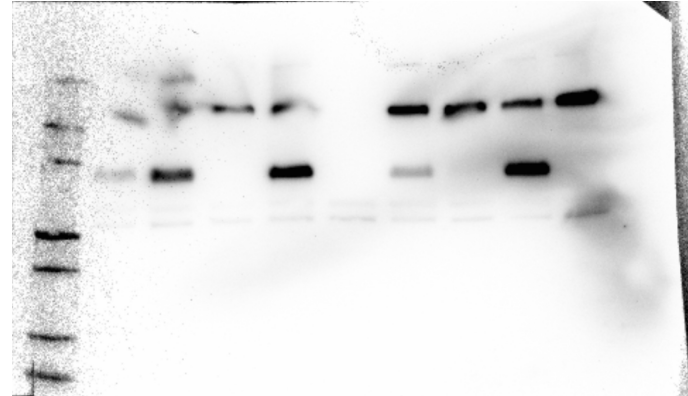

p55

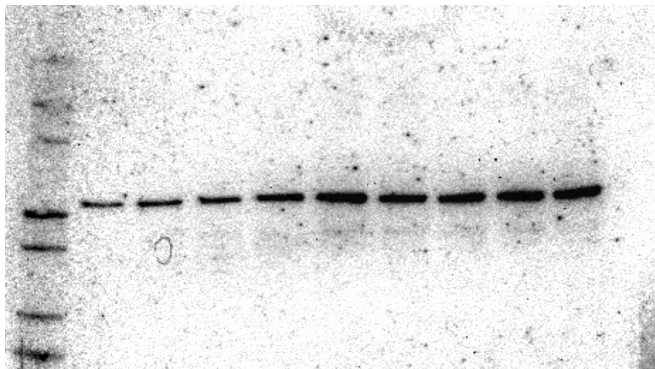

Actin

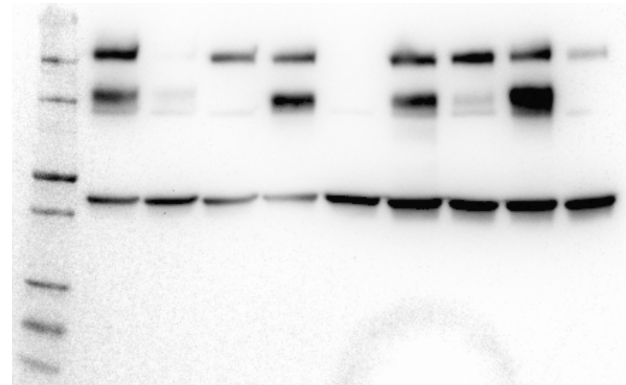

Supplement: Source Data Fig. 6 — Raw scans of blot for Fig. 6d. [file 41564_2022_1143_MOESM17_ESM.pdf]

Fig. 5 i

Full blot

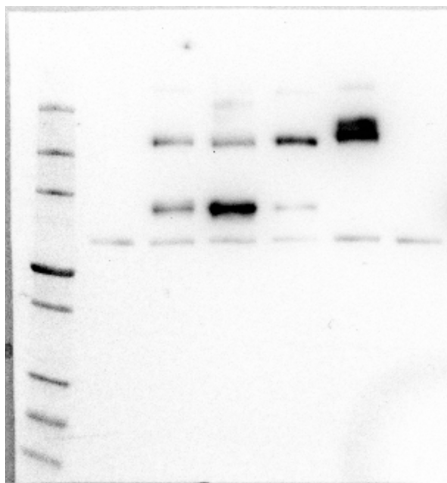

Actin Full blot

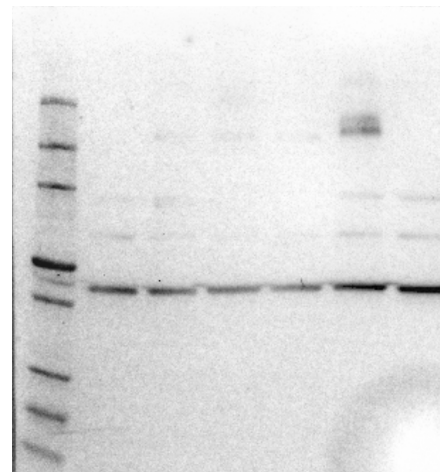

Supplement: Source Data Fig. 5 — Raw scans of blot for Fig. 5i. [file 41564_2022_1143_MOESM18_ESM.pdf]
